# Application of Pharmacokinetic-Pharmacodynamic Modeling to Inform Translation of *In Vitro* NaV1.7 Inhibition to *In Vivo* Pharmacological Response in Non-human Primate

**DOI:** 10.1007/s11095-020-02914-9

**Published:** 2020-09-04

**Authors:** Jeanine E. Ballard, Parul Pall, Joshua Vardigan, Fuqiang Zhao, Marie A. Holahan, Richard Kraus, Yuxing Li, Darrell Henze, Andrea Houghton, Christopher S. Burgey, Christopher Gibson

**Affiliations:** 1 Pharmacokinetics, Pharmacodynamics, and Drug Metabolism, Merck & Co., Inc., West Point, Pennsylvania USA; 2 In Vivo Neuropharmacology, Merck & Co., Inc., West Point, Pennsylvania USA; 3 MR-CT-US & Optical Imaging, Merck & Co., Inc., West Point, Pennsylvania USA; 4 Neuronal Signaling, Merck & Co., Inc., West Point, Pennsylvania USA; 5 Quantitative Biosciences, Merck & Co., Inc., West Point, Pennsylvania USA; 6 Discovery Chemistry, Merck & Co., Inc., West Point, Pennsylvania USA

**Keywords:** fMRI, NaV1.7, nociception, olfaction, PK-PD

## Abstract

**Purpose:**

This work describes a staged approach to the application of pharmacokinetic-pharmacodynamic (PK-PD) modeling in the voltage-gated sodium ion channel (NaV1.7) inhibitor drug discovery effort to address strategic questions regarding *in vitro* to *in vivo* translation of target modulation.

**Methods:**

PK-PD analysis was applied to data from a functional magnetic resonance imaging (fMRI) technique to non-invasively measure treatment mediated inhibition of olfaction signaling in non-human primates (NHPs). Initial exposure-response was evaluated using single time point data pooled across 27 compounds to inform on *in vitro* to *in vivo* correlation (IVIVC). More robust effect compartment PK-PD modeling was conducted for a subset of 10 compounds with additional PD and PK data to characterize hysteresis.

**Results:**

The pooled compound exposure-response facilitated an early exploration of IVIVC with a limited dataset for each individual compound, and it suggested a 2.4-fold *in vitro* to *in vivo* scaling factor for the NaV1.7 target. Accounting for hysteresis with an effect compartment PK-PD model as compounds advanced towards preclinical development provided a more robust determination of *in vivo* potency values, which resulted in a statistically significant positive IVIVC with a slope of 1.057 ± 0.210, R-squared of 0.7831, and *p* value of 0.006. Subsequent simulations with the PK-PD model informed the design of anti-nociception efficacy studies in NHPs.

**Conclusions:**

A staged approach to PK-PD modeling and simulation enabled integration of *in vitro* NaV1.7 potency, plasma protein binding, and pharmacokinetics to describe the exposure-response profile and inform future study design as the NaV1.7 inhibitor effort progressed through drug discovery.

**Electronic supplementary material:**

The online version of this article (10.1007/s11095-020-02914-9) contains supplementary material, which is available to authorized users.

## Introduction

There is consensus among the pharmaceutical industry that improved understanding of exposure–response relationships at an early stage in the drug hunting process will be important to inform lead optimization strategies and reduce attrition rates in the clinic (1,2). A retrospective analysis of Phase II trial data highlighted the importance of pharmacokinetic (PK) and pharmacodynamic (PD) principles underlying the “three pillars of survival” (3). At a high level, the three pillars of survival include being able to objectively measure and relate drug exposure, target engagement, and modulation of the desired pharmacology at the site of action. Ability to demonstrate all three pillars through the use of non-clinical and clinical information in conjunction with translational modeling approaches on a development candidate increased the probability of achieving a successful Phase II clinical outcome. One can speculate that having information and knowledge of the ‘3-pillars’ concepts on a development candidate can lead to better clinical hypothesis generation and early clinical development plans. Such knowledge enables the use of translatable biomarkers to facilitate clear quantitative go/no-go decisions and inform on key clinical study design questions such as dose selection and sampling times. Pharmacokinetic-pharmacodynamic (PK-PD) modeling and simulation is an important tool that can help quantitatively interrelate drug concentrations and their pharmacological effect. PK-PD models are particularly useful in characterizing exposure–response relationships where there are apparent disconnects because of temporal delays in drug action relative to drug exposure, a phenomenon referred to as hysteresis. For instance, a delay in onset and washout of action relative to drug exposure in the plasma could be due to slow distribution of the drug to and from its target site or an indirect relationship between exposure and response caused by biomarker synthesis and turnover rates or a function of the target binding kinetics (4).

Although *in vitro* pharmacological data in biochemical or cell systems are useful in drug discovery to rank the intrinsic potency of new compounds, it often has limited value in directly informing on the target exposure required for *in vivo* pharmacodynamics and efficacy. Underlying reasons for this apparent discrepancy between *in vitro* potency and *in vivo* pharmacology and efficacy can be multifactorial. Some common contributors are limited distribution from the blood to the target site and unaccounted for non-specific and plasma protein binding, both *in vitro* and *in vivo*. Thus, *in vitro* potency values need to be combined with appropriate knowledge of pharmacokinetics and drug distribution/binding to establish useful cross system translation such as *in vitro* to *in vivo* correlations (IVIVC) of pharmacological potency. PK-PD modeling enables the integration of all available information, from both *in vitro* and *in vivo* sources, to describe the exposure-response profile for a given drug. Such a mathematical model may also enable prospective translational simulations of the drug across biological systems, such as different assay platforms or different species.

Here we discuss the application of translational quantitative pharmacokinetic-pharmacodynamic modeling in the voltage-gated sodium ion channel NaV1.7 inhibitor drug discovery effort to address three key strategic questions.

1. How does *in vitro* potency translate to *in vivo* exposure for pharmacologic activity?
2. Is hysteresis observed *in vivo*, and if so, can it be accounted for with an appropriate PK-PD model?
3. What is the anticipated dose and regimen to achieve *in vivo* efficacy?

This work describes the *in vitro* to *in vivo* translation of NaV1.7 inhibition effect on olfaction in non-human primates (NHPs) and demonstrates the utility of simulation with the PK-PD model to inform study design for anti-nociceptive response assays.

There is genetic evidence supporting a role for the voltage-gated sodium ion channel NaV1.7 in sensitivity to pain (5–7). Loss-of-function mutations in the NaV1.7 gene (SCN9A) in human produces insensitivity to pain, while gain-of-function mutations have been associated with inherited pain syndromes (6,8). Furthermore, a number of drugs with sodium channel blocking activity, such as carbamazepine, lamotrigine, and several tricyclic antidepressants are already used in pain management (9–11). However, these drugs are not selective for the NaV1.7 isoform, and effectiveness is often limited by adverse central nervous system and cardiovascular side effects (9) which are attributed to the non-selective nature of these drugs. Selective inhibition of sodium channels specifically involved in pain pathways might have the potential to improve efficacy and safety (12). Therefore, NaV1.7 has become a promising target for pharmaceutical intervention for various human pain conditions (13).

**Fig. 1:**
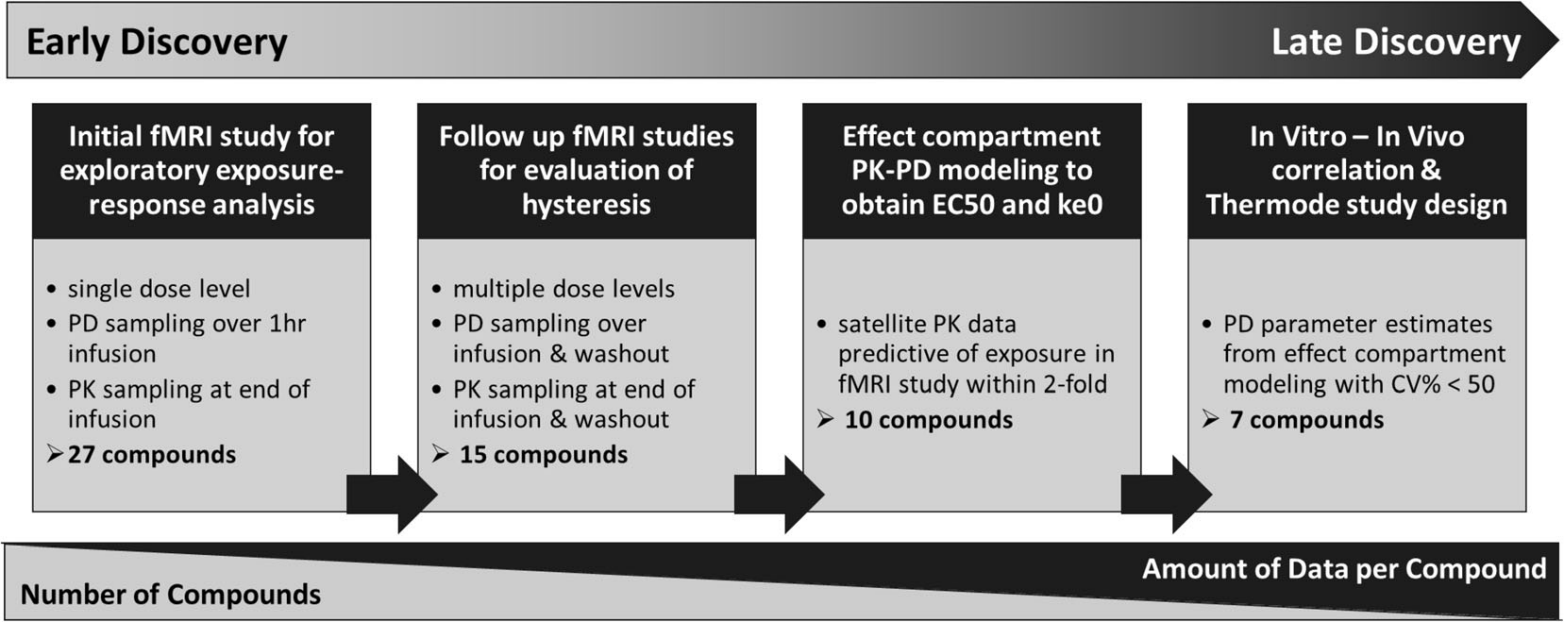
Schematic representation of the staged approach to exposure-response analysis in drug discovery. The Early Discovery phase primarily consists of many compounds with limited data available for each compound. Substantially fewer compounds progress into Late Discovery, accompanied by an increase in the amount of data available for each compound to evaluate the impact of dose and time dependency in PK-PD analysis.

The ability to recapitulate the human NaV1.7 loss-of-function phenotype with pharmacological inhibition of NaV1.7 channels has been demonstrated in a number of rhesus macaque models (manuscript in preparation). One model in particular leverages the phenomenon of anosmia (i.e. loss of the sense of smell) reported in NaV1.7 loss-of-function subjects. Humans with loss-of-function mutations in NaV1.7 are anosmic (14) suggesting that odor-detection may be a useful target modulation biomarker for NaV1.7 inhibitors. A functional magnetic resonance imaging (fMRI) technique which can non-invasively measure odor-induced olfaction signaling in the olfactory bulb (OB) was developed in NHPs (15). This technique was employed during drug discovery to measure treatment-mediated inhibition of odor-induced activation in the OB of rhesus macaques as a target modulation biomarker of *in vivo* NaV1.7 inhibition. PK-PD analysis of the data from fMRI olfaction studies was conducted in stages to characterize the *in vivo* potency for molecules with a range of *in vitro* NaV1.7 blocking potencies. Initial exploratory exposure-response analysis included 27 compounds for which fMRI olfaction data was obtained in an assay format with limited duration and dose levels, but it enables acquisition of data for multiple different compounds. A more robust PK-PD modeling approach was applied for a subset of 10 compounds which had a more extensive fMRI dataset consisting of longer duration of PD measurement and multiple dose levels along with PK data from satellite studies. Figure 1 outlines the data available and number of compounds at each stage of analysis as the project progressed through drug discovery. Modeling and simulation enabled quantitative translation of *in vitro* NaV1.7 potency and other salient information such as PK, to *in vivo* pharmacological response and subsequently informed the design of efficacy studies to demonstrate anti-nociceptive response to a noxious thermal stimulus in NHPs.

## Materials and Methods

### *In Vitro* Measurement of Rhesus NaV1.7 Inhibition

HEK293 cells recombinantly overexpressing rhesus NaV1.7 channels were used for patch clamp experiments. Test compounds were prepared as 10 mM stock solutions in DMSO. Ten concentrations of each compound were used to generate 10-point calibration curves and were prepared using the Echo acoustic liquid handling instrument and glass coated 384-well plates. The final DMSO concentration in each well was 0.3%. 384-well whole-cell NaV current recordings per Qchip were performed on the QUBE (16). Before addition of test compound, cells in each well on the Qchip were monitored for stability for at least 5 min. For each concentration, test compound was applied 3 times to each well on the Qchip to achieve equilibrium. Washout was performed to measure recovery of the sodium current. The following external and internal solutions were used: External solution: 150 mM NaCl, 5 mM KCl, 2 mM CaCl_2_, 1 mM MgCl_2_, 10 mM HEPES, 12 mM Dextrose, pH 7.3 with NaOH; Internal solution: 30 mM CsCl, 5 mM HEPES, 10 mM EGTA, 120 mM CsF, 5 mM NaF, 2 mM MgCl_2_, pH = 7.3 with CsOH.

The following pulse protocol was used. Cells were held at −90 mV. A train of 6 consecutive test pulses to −10 mV was applied at a frequency of 0.1 Hz in the absence and presence of test compound. Each test pulse was preceded by an 8 s prepulse to −115 mV. Cells in each Qchip well were exposed to a single test compound concentration for 5 min at −90 mV before inhibition was measured.

Percent inhibition of sodium channel current (*I*_*NaV*_) at each compound concentration was determined using Eq. 1.

1 $$ \% Inhibition\ of\ {I}_{NaV}=100\ast \left(\frac{{I_{NaV}}_{control}-{I_{NaV}}_{drug}}{{I_{NaV}}_{control}}\right) $$

*IC*_*50*_ values were calculated using a 4-parameter logistic function (Eq. 2) where *IC*_*50*_ = half maximal inhibitory concentration; *I*_*min*_ = minimal current (fixed to 0); *I*_*max*_ = maximal current (fixed to 100); *h* = Hill coefficient; *x* = compound concentration.

2 $$ f(x)={I}_{min}+\left(\frac{I_{max}-{I}_{min}}{1+{\left(\frac{IC_{50}}{x}\right)}^h}\right) $$

Results are expressed as geometric mean ± standard deviation.

### *In Vitro* Measurement of Plasma Protein Binding

The unbound fraction of compound in plasma from rhesus macaques was measured by equilibrium dialysis of 2.5 μM compound in 100% plasma against 100 mM PBS buffer using HT Dialysis plates (Model HTD96b) with 12–14 MWCO dialysis membranes. The plate was equilibrated for 4 h at 37°C in a humidified incubator with a 5% CO_2_ environment. The study samples were prepared for a protein precipitation extraction method by addition of acetonitrile containing a cocktail of internal standards (labetalol, imipramine, and diclofenac). The peak area ratio of test compound to internal standard in plasma and buffer were determined by ultra-high performance liquid chromatography (UPLC) coupled with a SCIEX API triple quadruple mass spectrometer. Chromatographic separation was performed using reverse phase LC gradient methods. The fraction unbound in plasma is the ratio of test compound/internal standard peak area ratio in buffer to that of test compound/internal standard peak area ratio in plasma.

### Animals

Rhesus macaques were the species of choice over rodents for the olfaction assay due to greater similarity to human, both anatomically and functionally, in the OB and olfactory tract (17–20). Rhesus macaques were also used for anti-nociceptive response assays because many compounds being investigated did not have sufficient potency against the rodent isoform of NaV1.7. Male and female rhesus macaques weighing 4–12 kg were used in the experiments. Individual animals were single- or pair-housed and maintained on a 12 h light cycle (06:30–18:30 h) with room temperatures maintained at 22 ± 2°C. Animals were fed their full daily regimen of food (Purina High Protein Monkey Diet no. 5045) prior to testing and water was available *ad libitum*. All monkeys were given a variety of fresh fruits and vegetables daily in addition to the standard food regimen. Principles from the Guide for the Care and Use of Laboratory Animals, National Institute of Health, and USDA were followed, and all protocols were approved by the Institutional Animal Care and Use Committee of Merck & Co., Inc., Kenilworth, NJ, USA.

### Functional Imaging of Olfaction by Cerebral Blood Volume fMRI in Rhesus Macaque

For the fMRI study, the detailed methods including animal preparation, experiment set-up, anesthesia protocol, odor stimulation, MRI measurement, and data analysis have been described previously (15). Briefly, each animal was initially sedated using a cocktail of ketamine (3 mg/kg, IM) and dexmedetomidine (40 μg/kg, IM) for catheterization and experimental set-up. The animal was then secured in the supine position in the MRI scanner, and the anesthesia changed to a continuous delivery of isoflurane (0.25%) and dexmedetomidine (IV infusion of 15 μg/kg/h). The USPIO contrast agent (Feraheme) was administered (10 mg/kg, IV) before the start of fMRI data acquisition to improve the fMRI sensitivity. For odor stimulation, isoamyl acetate (~2870 ppm) was introduced to the animal’s nostrils by automated opening of two valves at the inlet and outlet of a bubbling bottle containing the odor solution. The test compound solution was prepared immediately before the study. For different dose studies, test compound was dissolved in 30% captisol at various concentrations to make the total dose volume for each animal relatively constant (~8 mL/monkey). The solution was sonicated and filtered to ensure sterility. The fMRI measurements were performed on a 3-T, Siemens Trio system. A 16-channel head coil was used as the radiofrequency receiver. Scout images in three orthogonal directions were first acquired using fast low angle shot sequence. Based on the sagittal image, twenty-four consecutive axial slices were chosen for the fMRI study. T2*-weighted images were acquired using a single-shot gradient echo echo-planar imaging sequence: matrix size = 64 × 64, field of view = 12 × 12 cm^2^, slice thickness = 1.8 mm, repetition time = 3 s, and gradient echo time = 28 ms. The corresponding spatial resolution was 1.9 × 1.9 × 1.8 mm^3^, and the acquisition time for imaging the entire volume was 3 s. The stimulation paradigm: 1 min (baseline) + 1 min (odor stimulation) + 2 min (recovery). One single fMRI measurement required 4 min, with a total of 20 (baseline) + 20 (stimulation) + 40 (recovery) volume acquisitions. Fifteen fMRI measurements were made for each NHP per 1 h period. The compound or vehicle delivery was started after 1 h of data acquisition and was continuously infused for the following hour. In some experiments, data acquisition continued for up to 2 h after the end of compound infusion. Plasma samples were collected from each animal at the end of infusion and end of experiment to measure concentration of test compound.

Data was processed using Stimulate (21) and custom MATLAB routines (Mathworks, Natick, MA). The time courses of the fMRI signals in the OB were first obtained by averaging the time courses of all the activated pixels within the OB for each fMRI measurement, and the time courses from 3 consecutive fMRI measurements were further averaged. Therefore, fifteen fMRI measurements performed during each 1-h block of experiment yielded equivalent 5 fMRI responses. Each experimental session could be from 2 to 4 h in duration, resulting in 10 to 20 fMRI responses. The strength of each fMRI response was quantified by averaging the amplitudes of fMRI signals during the stimulation period. The inhibition of olfaction was expressed as percentage inhibition of the averaged fMRI responses during the 1-h period before compound delivery using Eq. 3.

3 $$ \% Inhibition=100\ast \left(\frac{{ fMRIres ponse}_{baseline}-{ fMRIres ponse}_{drug}}{fMRIres\mathrm{p}{onse}_{baseline}}\right) $$

**Fig. 2:**
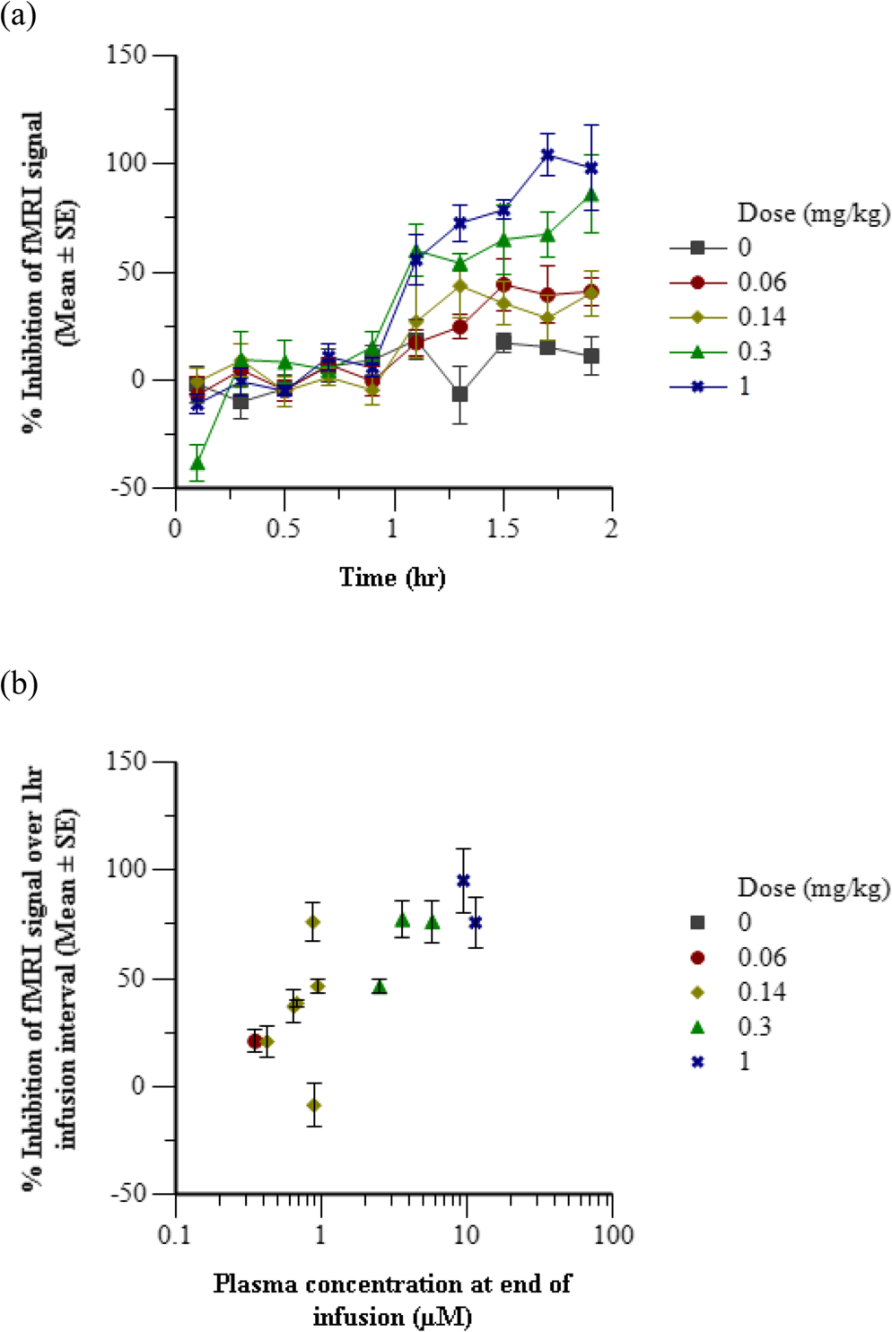
Inhibitory effects of representative Compound 24 on odor-induced olfaction in NHP. (**a**) Time course of average inhibition of fMRI signal for each dose group. Baseline was established for 1 h followed by a constant infusion for 1 h with Compound 24. (**b**) Average inhibition of fMRI signal over the 1 h infusion interval *versus* the plasma concentration at the end of the infusion in each animal. Error bars represent the standard error of the mean.

A total of 27 compounds plus a vehicle control were evaluated in the initial fMRI olfaction assays. Typical fMRI study design for each compound consisted of 1 to 3 dose groups with an average of 3 animals per group and 2 h of fMRI measurements, although a subset of 15 compounds had more extensive datasets from follow up fMRI olfaction assays with up to 7 dose groups, a range of 1 to 6 animals per group, and up to 4 h of PD measurments (Online Resource 1). Individual percent inhibition datapoints were aggregated in two different ways to enable visual representation of the average temporal PD profile and the exposure-response relationship. The arithmetic mean of percent inhibition from all animals within each treatment group was calculated at each time point for Fig. 2a. The arithmetic mean of percent inhibition from all time points during the 1 h of drug infusion was calculated for each individual animal in Fig. 2b.

### Rhesus Thermode Assay

Thermode testing was performed in NHPs as outlined in Vardigan *et al*. 2018 (22). Briefly, animals were chaired with their arms restrained against the front horizontal panel using umbilical tape. Veterinary wrap was applied to each wrist to prevent abrasive contact with tape, which was then tied around the wrists and affixed to a lower-front portion of the chair with sufficient slack to avoid distress and allow for visible withdrawal motions. Each animal’s hair on the underside of its forearms was shaved and the thermode stimulator was attached to shaved area of the left or right forearm by veterinary wrap. Test compound was dissolved in 0.9% NaCl/3% PEG3350/0.3% TWEEN80 and administered subcutaneously (SC) on the back at 0.2 mL/kg. Only 5 of these test compounds were ultimately included in the thermode assay, and each study consisted of 4 to 5 dose groups, including a vehicle control, with typically 6 to 8 animals per group (Online Resource 1). Thermal stimulation was delivered via Medoc Thermode software and triggered with an external handheld trigger. Four heat stimuli (44, 46, 48, or 50°C) were presented pseudo-randomly in six blocks. These temperatures were selected based on reported human pain thresholds with the same thermode device (23–26). Each stimulus was presented for 5 s, and stimuli were presented under a variable interval 22.5 s (range = 15–30 s). Each response was assessed using a three-point scale, where 0 = no response, 1 = response consisting of a single clear arm movement, 2 = multiple arm or body movements. Occasionally a score of 0.5 was used to indicate a very small or questionable response. Only animals exhibiting a minimum mean score of 1 at 48°C and 1.5 at 50°C under baseline conditions were selected for study. Plasma samples were collected from each animal immediately following thermode testing to measure concentration of test compound. The thermode response score was converted into percent of baseline by dividing the individual response score in the presence of drug at each dose level by the average baseline response score from vehicle treated animals. The arithmetic mean and standard error were then calculated for each dose group.

### *In vivo* PK Studies and Bioanalytical Methods

Pharmacokinetic studies of 13 test compounds were conducted in fasted male rhesus macaque via intravenous (IV) administration. Test compounds were administered at various doses ranging from 0.05 to 15 mg/kg with most compounds having just one dose level and two or three animals per dose group (Online Resource 1). Plasma samples were collected at multiple time points (typically nine samples) from 2 min up to 24 h post dosing and stored at −20°C until analysis. Compound 6 is the only compound which had satellite PK samples collected under the same conditions, dose levels, and time points as the fMRI study. The study samples and standard curve in control matrix were prepared for a protein precipitation extraction method by addition of 0.1% formic acid in acetonitrile containing a cocktail of internal standards (labetalol, imipramine, and diclofenac). Concentrations of test compound in plasma were quantified using UPLC system coupled with a SCIEX API triple quadruple mass spectrometer. Chromatographic separation was performed using reverse phase liquid chromatography gradient methods. The concentrations of test compound in the samples were determined using MultiQuant 3.0.1 based on standard curves prepared in corresponding control matrices.

### Modeling & Simulation

The E_max_ model is a common PK-PD model used to describe pharmacological responses, where *E* is the pharmacological effect, *C* is the drug concentration, *E*_*max*_ is the maximum achievable effect, *E*_*0*_ is the effect at baseline, and *EC*_*50*_ is the concentration producing half of the maximum effect (Eq. 4).

4 $$ E={E}_0+\frac{E_{max}\ast C}{EC_{50}+C} $$

This approach assumes a direct relationship with no observable hysteresis between plasma concentration and inhibition of olfaction. Preliminary exposure-response analysis of the fMRI data involved an E_max_ model fit using data pooled across different NaV1.7 inhibitors and studies. The modeling was conducted using a naïve-pooled algorithm with additive residual error in Phoenix 64 software (Build 7.0.0.2535, Certara, Princeton, NJ). Plasma concentrations (*C*_*p*_) measured at the end of the infusion and at the end of the study for each compound were multiplied by the measured fraction unbound in rhesus plasma (*f*_*u,p*_) and then divided by the measured *in vitro* rhesus NaV1.7 *IC*_*50*_ value. Therefore, the exposure term *C* in Eq. 4 becomes a derived dimensionless value for unbound plasma concentration normalized to *in vitro* potency as in Eq. 5.

5 $$ E={E}_0+\frac{E_{max}\ast \left(\frac{C_p\ast {f}_{u,p}}{in\ vitro\ {IC}_{50}}\right)}{EC50+\left(\frac{C_p\ast {f}_{u,p}}{in\ vitro\ {IC}_{50}}\right)} $$

While the pooled cross-compound approach was useful for obtaining initial estimates of the *in vitro* to *in vivo* relationship, it did not account for compound specific parameters which lead to observed hysteresis. An empirical model that is often applied to temporal delays believed to be related to distribution to the target site is the effect compartment model, which is also referred to as an indirect link or biophase distribution model (27,28). The model contains a hypothetical compartment for concentrations at the site of effect (*Ce*) with a first-order distribution rate constant *ke0* (Eq. 6). The PD model structure is a modification of the E_max_ model in Eq. 1 to incorporate concentrations in the effect compartment (*Ce*) as the exposure term which drives the response (Eq. 7). The PD model fitting was conducted for individual animals using a naïve-pooled algorithm with additive residual error in Phoenix 64 software (Build 7.0.0.2535, Certara, Princeton, NJ).

6 $$ \frac{dCe}{dt}= ke0\ast Cp- ke0\ast Ce $$

7 $$ E={E}_0+\frac{E_{max}\ast Ce}{EC50+ Ce} $$

Characterization of the plasma concentration over time is required for application of an effect compartment model. However, frequent sampling for PK during the olfaction study was not feasible since the animal was isolated inside the fMRI machine. Therefore, satellite PK studies in rhesus macaques were used to enrich the pharmacokinetic information needed for the PK-PD modeling. Satellite PK data and fMRI measurements in the washout period was not available for all 27 compounds in the fMRI dataset, so PK modeling to support effect compartment modeling was only applied to a sub-set of 13 compounds. A two-compartment PK model structure with IV administration (Eq. 8) was fit to the plasma concentration-time data from satellite studies using an IV bolus or IV infusion dosing paradigm. A population model with random effects on all parameters and a log-additive residual error was fit using a First Order Conditional Estimation Lindstrom-Bates algorithm. Parameter value estimates of clearance (*CL*), distribution clearance (*CL2*), volume of the central compartment (*V*), and volume of the peripheral compartment (*V2*) were obtained for each test compound. Visual predictive checks and CV% on parameter estimates were used to evaluate the model fits.

8 $$ {\displaystyle \begin{array}{c}V\ast \frac{dC}{dt}=- CL\ast Cp- CL2\ast Cp+ CL2\ast C2\\ {}V2\ast \frac{dC2}{dt}= CL2\ast Cp- CL2\ast C2\end{array}} $$

Population estimates of *CL*, *V*, *V2*, and *CL2* from the satellite PK studies were subsequently used to simulate the concentration-time profile that would be achieved with the IV bolus followed by IV infusion regimen at each dose level tested in the fMRI studies. The measured plasma concentrations at the end of the infusion and at the end of the experiment from the fMRI studies were used to qualify the PK model obtained from fitting the IV bolus data. Compounds were excluded from further analysis if the simulated plasma concentrations were not within 2-fold of the mean observed plasma concentrations. While the source of each missed PK prediction was not interrogated, some potential causes include dose non-linearity, effects of anesthesia, or gender differences between satellite PK and fMRI studies. This PK prediction performance criteria further reduced the number of compounds to a subset of 10 in the effect compartment PK-PD modeling.

The resulting PK and PD parameter estimates were then used to predict the dose anticipated to demonstrate inhibition of withdrawal responses to noxious stimuli in the NHP thermal stimulus assay following SC administration and to design the preclinical studies so that appropriate sampling times were used to capture the peak of effect. Simulations applied a two-compartment PK model structure with extravascular administration and an assumption that SC absorption was rapid (*ka* = 1) and complete (bioavailability = 100%). The assumptions regarding absorption properties were based on pharmacokinetic studies of structurally similar compounds in NHP which displayed rapid and near complete bioavailability following SC administration (data not shown).

## Results

### In Vitro Results

The inhibitory *in vitro* potency of 27 compounds was measured on rhesus NaV1.7 channels stably expressed in mammalian cells. The measured *IC*_*50*_ values are summarized in Table I along with measured fraction unbound in rhesus plasma. Intrinsic *in vitro* potencies of test compounds covered a wide range encompassing two orders of magnitude, ranging from 0.1 to 21 μM.

**Table I:** *In vitro* Fraction Unbound in Rhesus Plasma and Intrinsic Rhesus NaV1.7 Potency

Test compound	*f*_*u,p*_	*IC*_*50*_ (μM)
1	0.118	18.2
2	0.001	6.25
3	0.106	3.04
4	0.069	20.9
5	0.066	2.56
6	0.074	0.348
7	0.138	1.30
8	0.078	2.05
9	0.080	3.55
10	0.019	5.10
11	0.053	0.806
12	0.048	0.851
13	0.097	0.667
14	0.049	0.328
15	0.051	0.387
16	0.042	0.162
17	0.082	2.10
18	0.106	0.334
19	0.178	1.83
20	0.008	1.59
21	0.251	0.377
22	0.341	1.39
23	0.173	1.12
24	0.148	0.103
25	0.146	0.484
26	0.048	0.994
27	0.070	0.479

### fMRI Results

To determine modulation of pharmacology in rhesus macaques, the treatment mediated effect on odorant-induced olfaction in the OB of rhesus monkeys was measured by fMRI. Vehicle and a dose range of each of the 27 test compounds was studied in rhesus macaque monkeys. Odor-induced olfaction signaling was robustly observed in the OB of vehicle-treated NHPs and test compounds showed a concentration-dependent inhibition of the fMRI response. Fig. 2a shows the mean temporal profiles of the inhibition of fMRI responses for all treatment groups with representative test compound 24 for which a robust PK and PD dataset was acquired at multiple dose levels as the compound progressed through each stage of analysis. Given the administration paradigm of bolus followed by continuous infusion, the plasma concentration was assumed to be relatively constant over the entire hour post-dose, and this assumption was supported by subsequent PK modeling. This assumption allowed for an exposure-response relationship to be evaluated between the plasma concentration of test compound at the end of infusion and the average fMRI inhibition during infusion. Figure 2b is a typical representation of the fMRI data as average percent inhibition over the infusion interval *versus* the test compound concentration in plasma at the end of infusion.

### IVIVC with Cross Compound Exposure-Response Analysis

**Fig. 3:**
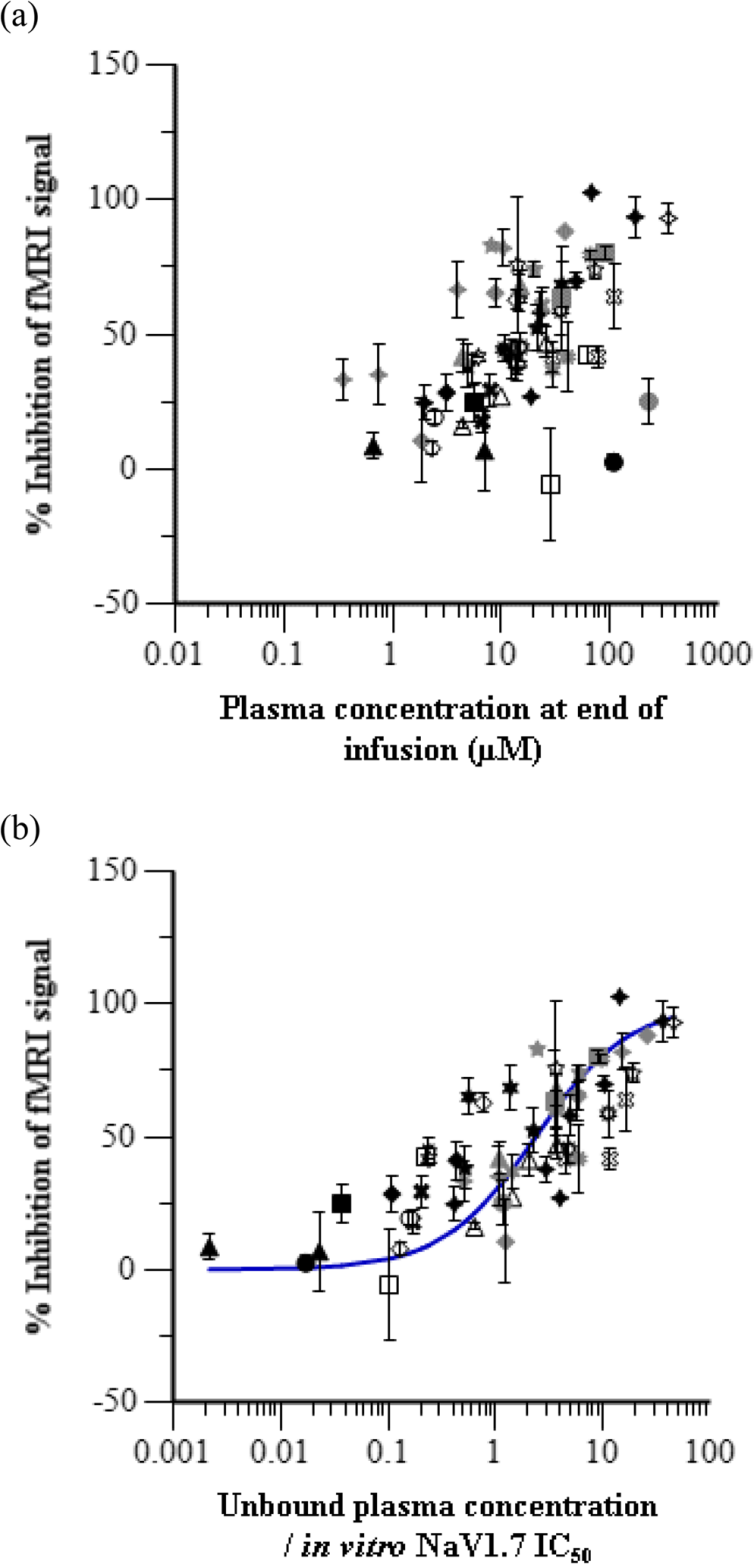
Pooled compound exposure-response in rhesus olfaction assay. Average inhibition of fMRI signal during the infusion interval at each dose level *versus* (**a**) the average *total* plasma concentration and (**b**) the average *unbound* plasma concentration *normalized by in vitro IC*_*50*_ for each test compound. Symbols represent observed data and error bars indicate standard error of the mean. The line is an overlay of the fitted model prediction.

The average percent fMRI inhibition and plasma concentration across individual animals within each dose group for each test compound was determined to enable comparison of the exposure-response relationship across different NaV 1.7 inhibitors. Figure 3a is an overlay of the average percent fMRI inhibition during the infusion interval at each dose level *versus* the average total plasma concentration at the end of the infusion for each of the 27 test compounds. A range of exposure-response curves was evident and is consistent with the range of *in vitro* potency values measured against rhesus NaV1.7. Correction of the exposure for plasma protein binding followed by normalization of the unbound plasma concentrations to the intrinsic *in vitro* potency for each test compound resulted in a collapse of this large apparent *in vivo* potency range to a narrower translational exposure-response profile with the exposure parameter represented as a dimensionless ratio of the unbound concentration divided by the *in vitro* rhesus NaV1.7 potency (Fig. 3b).

A simple E_max_ model was applied to characterize the relationship shown in Fig. 3b according to Eq.5. The estimated *EC*_*50*_ value is 2.35 ± 0.42, and *E*_*0*_ and *E*_*max*_ were fixed at 0 and 100%, respectively. Since the plasma concentrations are corrected for plasma protein binding and expressed relative to *in vitro* potencies, the *EC*_*50*_ parameter should be interpreted as the fold over *in vitro* potency that needs to be achieved in plasma, based on unbound concentrations, to elicit a 50% effect. The estimated *EC*_*50*_ parameter is in effect an *in vitro* to *in vivo* scaling factor.

### Effect Compartment PK-PD Modeling to Account for Hysteresis

**Fig. 4:**
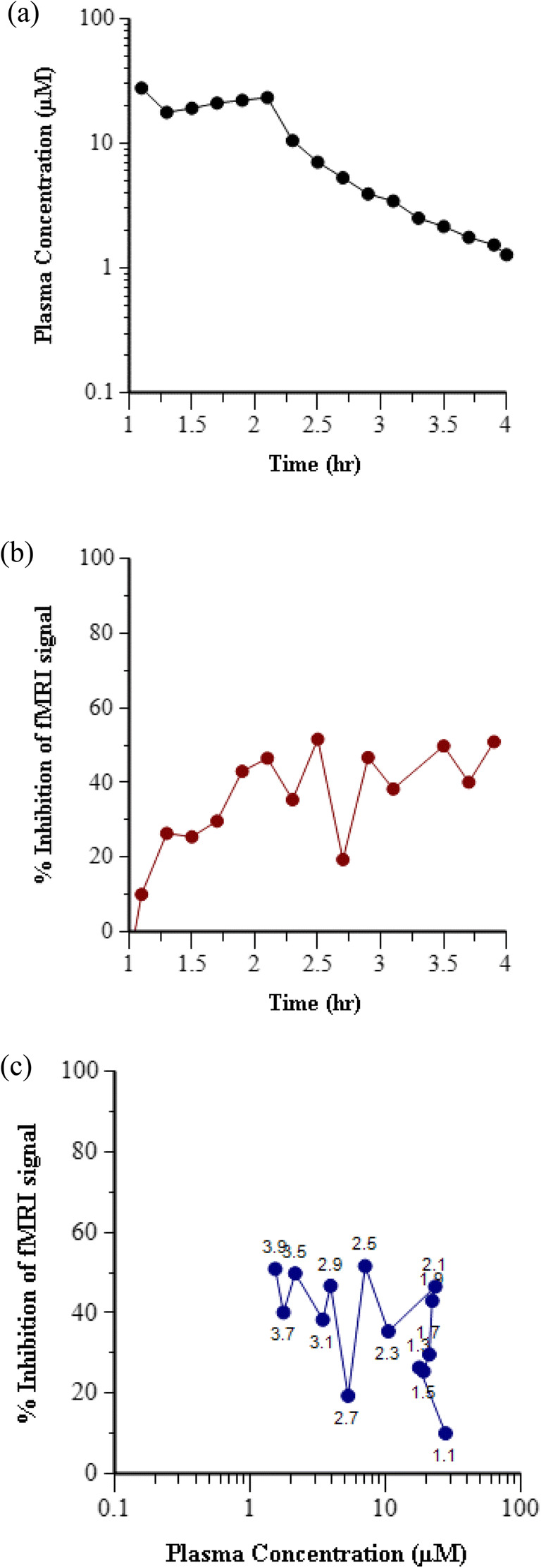
Temporal delay in effect relative to exposure in rhesus olfaction assay for representative Compound 6. (**a**) Time course of Compound 6 concentration in plasma following IV bolus plus 1 h infusion at a dose of 5 mg/kg in rhesus. (**b**) Time course of inhibition of fMRI signal following IV bolus plus 1 h infusion of 5 mg/kg Compound 6. (**c**) Hysteresis loop in the plot of inhibition of fMRI signal *versus* Compound 6 concentration in plasma at each time point. Data labels indicate the time in hours after start of fMRI data acquisition.

Figure 4 depicts the measured plasma concentration over time (PK) and percent change in fMRI response over time (PD) for Compound 6 at a single dose level for which both plasma concentration and fMRI measurements were obtained at the same time points throughout the 1 h IV infusion and 2 h washout period. Due to the IV bolus followed by IV infusion dosing paradigm of this study, the plasma concentrations remained relatively constant over the first hour of the study, then concentrations declined over the next two hours after the infusion ended (Fig. 4a). Despite the constant plasma concentration over the first hour, the percent change in fMRI response was slightly delayed and was observed to continue increasing over the first hour. While plasma concentrations were declining after the end of infusion, the effect level remained relatively constant (Fig. 4b). Thus, a time delay in both the onset of effect and the washout of effect relative to plasma exposure was evident from this data. When this data was plotted as change in fMRI response *versus* concentration at each time interval, a counterclockwise hysteresis loop was observed (Fig. 4c). At the early timepoints (1.1 to 2.1 h), while the plasma concentrations remained the same, the effect was increasing, and then after infusion (2.2 to 3.9 h) when the plasma concentrations were declining, the effect level remained high.

**Fig. 5:**
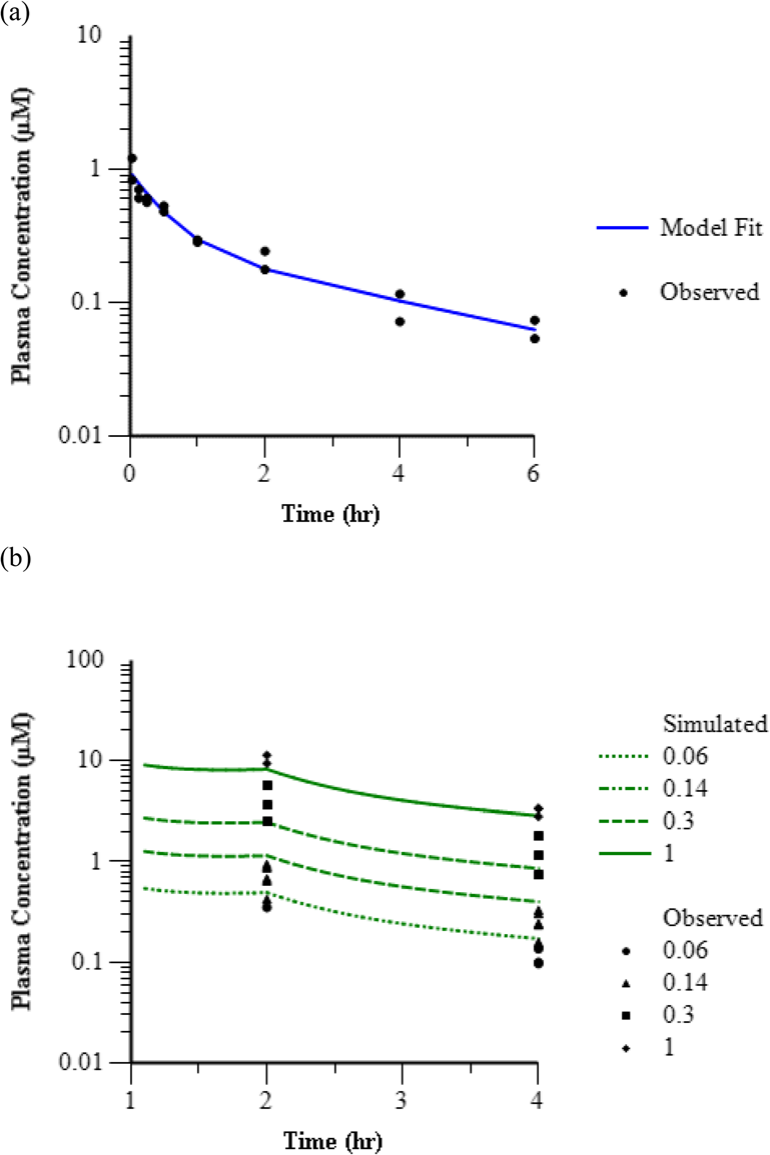
Modeling and simulation of plasma concentration over time for representative Compound 24. (**a**) Overlay of fitted model prediction (line) and observed (symbols) plasma concentration following IV bolus administration in rhesus satellite PK study at 0.05 mg/kg. (**b**) Overlay of model simulation (lines) and observed (symbols) plasma concentration following IV bolus plus 1 h infusion in rhesus olfaction assay at 0.06, 0.14, 0.3, and 1 mg/kg.

**Fig. 6:**
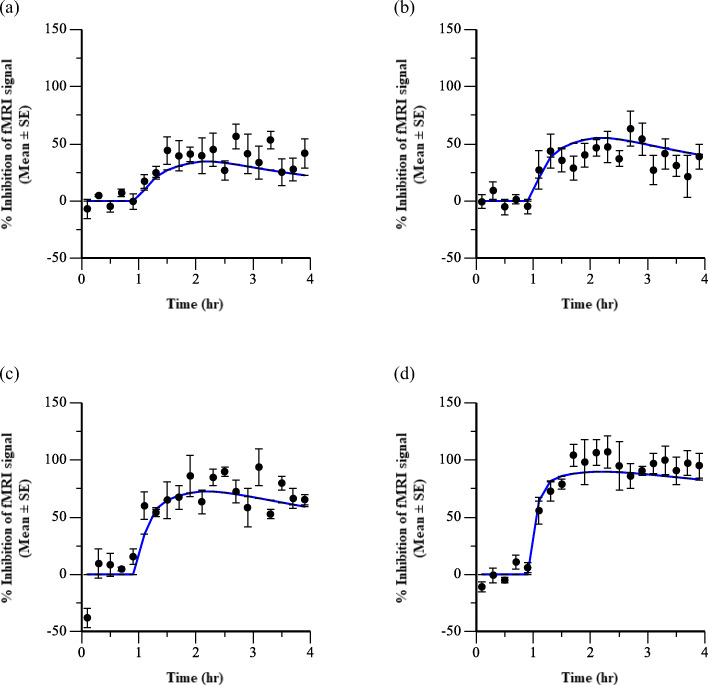
Time course of treatment mediated inhibition of fMRI signal with representative Compound 24. Symbols represent observed data (Mean ± SE) and lines are fitted effect compartment model predictions. Each panel displays a different dose of Compound 24 (**a**) 0.06 mg/kg (*N* = 3), (**b**) 0.14 mg/kg (*N* = 6), (**c**) 0.3 mg/kg (N = 3), and (**d**) 1 mg/kg (N = 3).

An example of the satellite PK modeling approach to characterize the plasma concentration over time in PD studies is demonstrated in Fig. 5 with representative compound 24. Figure 5a shows the model fit of satellite IV bolus PK data, and Fig. 5b shows the simulated plasma concentration over time under the dosing paradigm used in fMRI studies. The observed plasma concentration data from the end of infusion and end of study samples are overlaid on the plot as a visual predictive check and show a reasonable concordance. Satellite PK model fits and visual predictive check of simulated IV infusion doses of all compounds are included in Online Resource 2. From the 15 test compounds with fMRI olfaction data including measurement of PD washout, only 10 compounds had adequate PK model parameter fits from satellite PK data to support accurate simulation of the PK profile following IV bolus plus IV infusion dosing paradigm. Therefore, only for those 10 compounds, the PK parameters were fixed to the estimates from satellite PK modeling and used in the effect compartment PK-PD model applied to the percent change in fMRI response over time to account for hysteresis and obtain *EC*_*50*_ estimates. Similar to the initial model used to evaluate exposure-response, a simple E_max_ model with the *E*_*0*_ fixed at 0 and the *E*_*max*_ fixed at 100% was used to describe the PD within the effect compartment structure. The parameter estimates obtained from the model fit were *EC*_*50*_ and *ke0* (Table II). Figure 6 is an overlay of the observed and model fit of the PK and PD curves for a representative compound (Compound 24). Online Resource 2 depicts the overlay of mean observed and model fit PD curves for all 10 compounds included in the effect compartment analysis.

**Table II:** Parameter Value Estimates, Standard Errors (SE), and Coefficient of Variation (CV%) from Effect Compartment PK-PD Model Describing Inhibition of Rhesus Olfaction

Test Compound	*EC*_*50*_ (μM)			*ke0* (hr^−1^)		
	Estimate	SE	CV%	Estimate	SE	CV%
6	10.21	1.60	16	2.20	0.68	31
13	13.66	2.97	22	3.22	1.02	32
14	11.79	2.55	22	1.14	0.25	22
16	1.62	1.33	82	0.66	0.25	38
18	5.97	0.86	14	1.39	0.36	26
21	1.25	0.45	36	0.62	0.42	67
22	3.11	0.66	21	3.74	1.87	50
24	0.78	0.18	23	1.65	0.32	20
25	1.40	0.33	23	0.74	0.34	46
27	1.24	1.19	95	0.55	0.51	93

### In *Vitro* to *In Vivo* Translation of NaV1.7 Modulation

**Fig. 7:**
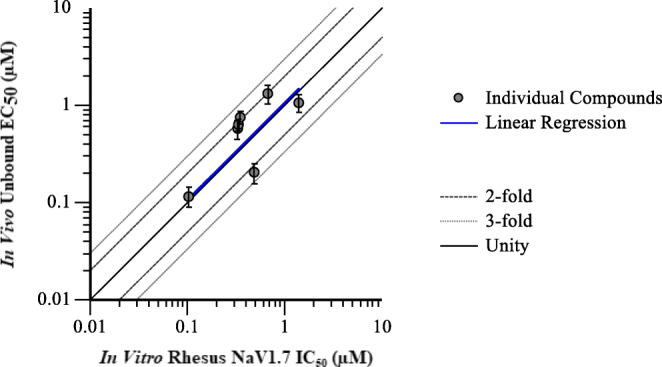
Correlation of *in vivo* unbound *EC*_*50*_ from effect compartment model of inhibition of olfaction with *in vitro* intrinsic NaV1.7 *IC*_*50*_. Symbols represent individual compound results, and the dashed line indicates unity. The solid line is a linear regression of the data points with an R-squared of 0.7831 and a slope of 1.057.

The *in vivo EC*_*50*_ estimates from effect compartment PK-PD model fitting for each individual test compound were corrected for free fraction in plasma to calculate an unbound *EC*_*50*_ estimate. The *in vivo* unbound *EC*_*50*_ was then compared to the *in vitro* rhesus NaV1.7 *IC*_*50*_ to evaluate the *in vitro* to *in vivo* correlation (Fig. 7). Only 7 of the 10 test compounds with sufficient confidence in the estimated *in vivo EC*_*50*_, defined as fitted *EC*_*50*_ and *ke0* CV% < 50, were included in the correlation analysis. The *in vivo* unbound *EC*_*50*_ correlated with the *in vitro* NaV1.7 *IC*_*50*_ with an R-squared value of 0.7831, a slope of 1.057 ± 0.210, and a *p* value of 0.006.

### Informing Study Design with Translational Model Simulation

**Fig. 8:**
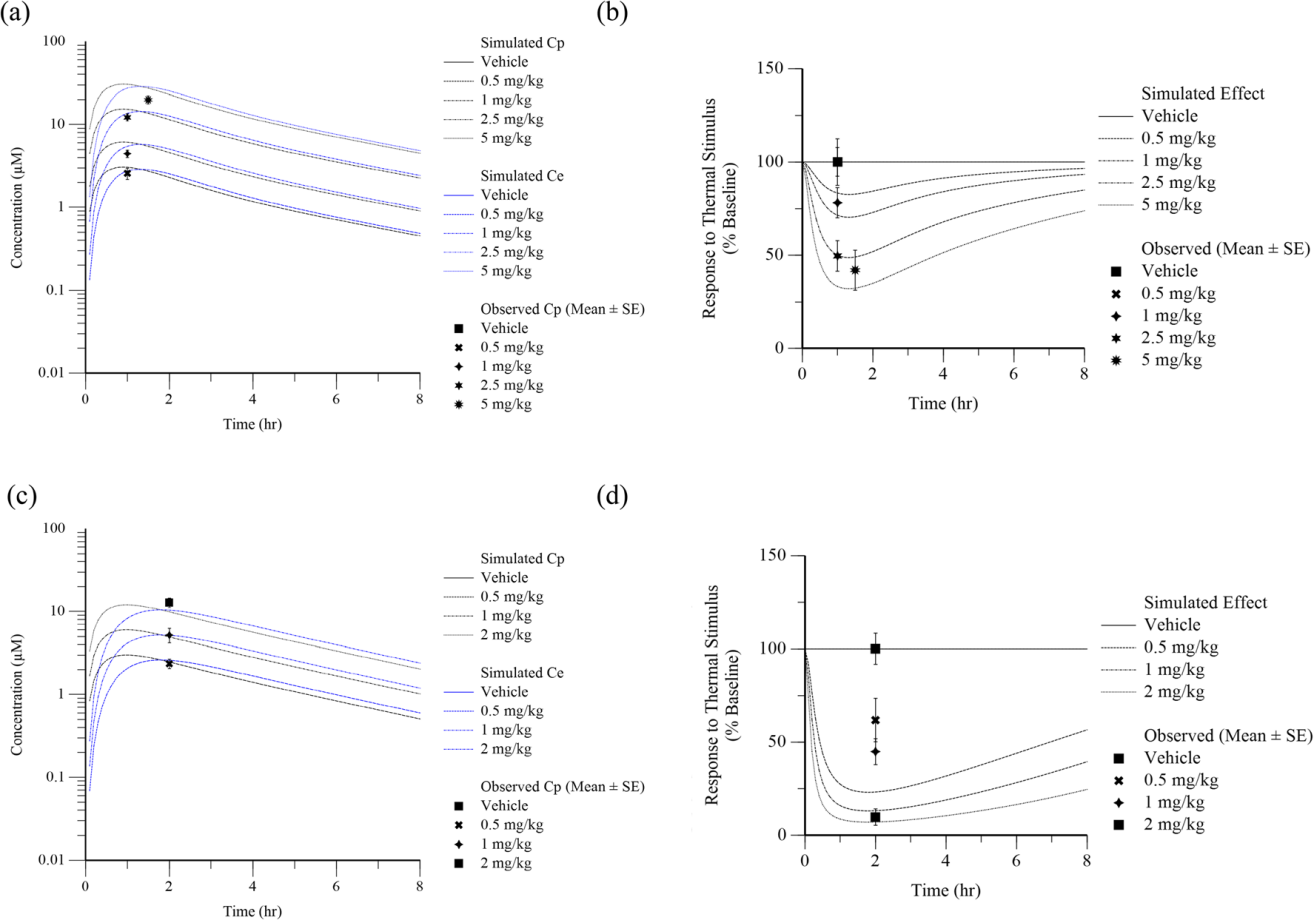
Time course of simulated (lines) and observed (symbols) concentration and nociceptive response for representative Compounds 13 and 24 following subcutaneous administration in the rhesus thermode assay. Top panel is Compound 13 data and bottom panel is Compound 24 data. Plots on the left (**a**, **c**) display concentration over time in plasma (black) and effect compartment (blue) and plots on the right (**b**, **d**) display effect over time in the rhesus thermode assay. Different shapes and line patterns indicate different doses as indicated in the figure legend.

An understanding of the exposure-response relationship and temporal delays for a given test compound against the NaV1.7 target in the fMRI olfaction assay enabled *a priori* extrapolation to other assays evaluating NaV1.7 pharmacology. An assay that was used to evaluate effectiveness for analgesic potential is the NHP thermode assay. Figure 8 shows the prospective simulated concentration over time (a,c) and effect over time (b,d) of two representative test compounds (Compound 13 and Compound 24) at various doses using the PK and PD parameters estimated from PK-PD modeling of the fMRI data. These simulations informed dose selection and sample time in the thermode assay. The measured concentration and percent effect at a single time point targeting Tmax in the effect compartment following SC administration of the proposed doses were overlaid in the plot as a visual predictive check. Simulated exposure-response for Compound 13 was highly predictive of the observed exposure-response in the NHP thermode assay. Compound 24 simulated exposure-response was approximately 4-fold left-shifted (more potent) than was actually observed in the NHP thermode assay. Compound 13 and 24 were selected to demonstrate the range of predictive performance using this approach, and Online Resource 3 shows the overlay of mean ± standard error of observed concentration and percent effect with model simulated curves for all 5 compounds for which NHP thermode assay data was available.

## Discussion

It is well accepted in the pharmaceutical industry that understanding of the exposure-response relationship of a compound for its intended target is a critical component of drug discovery. PK-PD modeling and simulation of preclinical data in drug discovery enables integration of the drug and system specific properties governing the observed PD profile, such as intrinsic potency against the target, *in vitro* and *in vivo* protein binding, PK, and distribution to the site of action. Beuters *et. al.* provided an overview of these key concepts and highlighted the impact in the drug discovery space with a few relevant examples (29). One compelling example is the robust 1:1 IVIVC established for β-secretase inhibitors in mice (R-squared = 0.85, *n* = 10, *p* < 0.01), which incorporated unbound fraction in brain along with turnover kinetics of the Amyloid β biomarker to account for hysteresis (30). This is contrasted with an earlier effort by Wood *et. al.* to define the *in vitro* to *in vivo* relationship for β-secretase inhibitors by pooling single dose level data from a single time point across 134 compounds in rats using total plasma concentrations (31). While a significant correlation was observed in the pooled compound approach, the regression was not very precise (R-squared of 0.26), and accounting for unbound fraction and time dependencies would likely have improved the correlation and understanding of magnitude of shift between *in vitro* and *in vivo* potency.

The work described here demonstrates the utility of translational PK-PD modeling and simulation of preclinical data in the early stages of drug discovery to evaluate *in vitro* to *in vivo* potency correlation, inform design of preclinical efficacy studies, and establish a translational approach which could be applied to inform human efficacious dose for NaV1.7 inhibitors. Similar approaches to exposure-response analysis and IVIVC have been described for the NaV1.7 target previously. Kalezic *et. al.* described application of PK-PD modeling of ectopic activity in spinal nerve ligated rats which incorporated an effect compartment to account for hysteresis and plasma protein binding to calculate unbound *in vivo* potency (32). A robust correlation (R-squared = 0.86) was established between the unbound *in vivo IC*_*50*_ and *in vitro* NaV1.7 *IC*_*50*_ with a 2 to 5-fold shift observed between *in vitro* and *in vivo* potency. The authors discuss the impact of this analysis on reducing the amount of *in vivo* efficacy screening required to optimize novel NaV1.7 inhibitors as a result of increased confidence in the *in vitro* to *in vivo* translation enabling only promising compounds based on *in vitro* potency and early PK analysis to be characterized in animal models. The article also attempts to relate clinically efficacious unbound exposures for marketed non-selective sodium channel inhibitors to *in vivo IC*_*50*_ for ectopic activity in the rat while acknowledging that the correlation relies heavily on one compound in the analysis and that additional data with more potent and selective NaV1.7 inhibitors would be needed to validate the relationship. The IVIVC established for olfaction as a marker of target pharmacology in non-human primates builds upon the existing knowledge from rodent models of ectopic activity. In addition, both the fMRI assay for olfaction and the thermode assay for nociception have the potential to be directly translated to clinical assays for NaV1.7 target modulation in healthy subjects.

The complexity of PK-PD model structure and precision of parameter estimates should be fit for purpose to answer the key question or test the hypothesis for which it was designed. At early stages of drug discovery, a pooled cross-compound approach to analysis is useful for obtaining initial estimates of the pharmacological *in vitro* – *in vivo* correlation (IVIVC) for chemical series and informs compound selection and progression in a drug discovery program. Pooled compound PK-PD analysis can often overcome the limitation of sparse *in vivo* data for individual compounds in the early stages of drug discovery by allowing multiple studies to be analyzed together. As demonstrated in Fig. 3, incorporation of compound and species-specific factors such as differences in plasma protein binding and intrinsic potency on the pharmacological target can improve the exposure-response relationship relative to the relationship that can be observed by using plasma concentration alone. In the case of our analysis, the measured *in vitro* potency was assumed to be the intrinsic potency against the target. Since the incubation media did not contain plasma or serum, no correction was made to account for protein binding in the *in vitro* system. Other compound specific parameters, such as the kinetics of distribution to the site of action, may still confound our understanding of the *in vitro* to *in vivo* translation due to hysteresis. Lack of accountability for compound differences in hysteresis undoubtedly contributed to the variability in our cross-compound exposure-response analysis.

As mentioned previously, there are multiple potential mechanisms for time dependency in PK-PD relationships, one of which is the rate of distribution to the site of action. For this particular target (NaV1.7), the site of action is unmyelinated sensory afferent nerves (33). Test compounds in this dataset have poor passive permeability, and therefore may have relatively slow distribution out of the vasculature and into the nerve tissue. The degree of temporal delay observed was not the same for all compounds, which suggests that the cause of hysteresis is a compound dependent property rather than a function of the pharmacology. Utilization of the effect compartment PK-PD model allows us to account for compound differences in rate of distribution to the target site when direct measurement of drug at the target site is not feasible. Application of this approach for our analysis successfully captured the effect-time profile as depicted in Fig. 7 and provided a more robust *in vivo* potency estimate for individual compounds. Comparison of the unbound *in vivo EC*_*50*_ estimate from PK-PD modeling with the *in vitro* rhesus NaV1.7 *IC*_*50*_ demonstrated a good correlation with a slope near unity, indicating that *in vivo* unbound *EC*_*50*_ was equivalent to *in vitro IC*_*50*_. The pooled compound analysis estimated that *in vivo* unbound *EC*_*50*_ was 2.4-fold greater than *in vitro IC*_*50*_. Therefore, individual compound PK-PD modeling with incorporation of an effect compartment to account for hysteresis improved our understanding of the *in vitro* to *in vivo* correlation.

Robust characterization of the plasma PK profile is critical to successful application of an effect compartment model. This requires a rich concentration-time dataset, ideally in the same animals as the effect-time dataset. However, frequent sampling of plasma concentrations during the fMRI experiments was not feasible. Therefore, when plasma concentration-time data from satellite studies was available, it was utilized to obtain the PK parameters. Although often necessary in preclinical studies where PK sampling is limited, use of satellite PK can introduce another source of variability in the estimate of *in vivo* potency. In this analysis we were assuming that the PK parameters following bolus IV administration to conscious animals at one dose level were representative of the PK parameters following IV infusion to anesthetized animals at a different dose level. Our fMRI experiments included limited sampling of PK at the end of the infusion as well as the end of the study, and this data was used to verify that the simulated PK profiles based on satellite studies were consistent with the observed data in fMRI experiments as in Fig. 6b.

While the fMRI olfaction assay was useful to evaluate target modulation and establish IVIVC, it does not directly inform on the efficacy of NaV1.7 inhibitors for antinociception. Therefore, compounds of interest were tested for antinociceptive potential to a noxious thermal stimulus in NHPs. Prospective simulation of the effect-time profile in rhesus thermode studies based on the PK-PD parameters estimated from modeling of the fMRI data enabled model informed selection of appropriate dose levels and sampling times. The ke0 parameter is particularly important when trying to select sampling time points which capture the maximum effect. Smaller *ke0* values result in a greater shift between Tmax observed in plasma concentration and Tmax predicted in the effect compartment. As demonstrated in Fig. 8, and Online Resource 3, the duration of delay in maximum effect relative to maximum plasma concentration ranged from approximately 0.5 h to 1.5 h. As evident in Fig. 8, the prediction of potency in the thermal nociception model was better for some compounds than others. A couple of critical assumptions were made in translating PK-PD from olfaction to nociception. The first was that the level of target engagement (NaV1.7 inhibition) required to block nociceptive signaling was the same as that for prevention of olfaction signal. Ex vivo electrophysiology studies in SCN9A KO olfactory sensory neurons demonstrated that the NaV1.7 mediated mechanism of action for anosmia is consistent with the mechanism responsible for absence of nociceptive perception in loss-of-function patients (14,34,35). Since the role of NaV1.7 in both processes involves propagation of action potentials from the sensory neuron to transmit signal to second order neurons, it seems reasonable that the same level of NaV1.7 inhibition would be required to block both physiological responses. The second assumption was that the rate of distribution to the site of action for olfaction (olfactory epithelium) would be the same for peripheral nerves of the forearm. However, the sensory cells of the olfactory system, olfactory receptor neurons (ORNs), are unique in that their cell bodies reside in the olfactory epithelium, while their dendrites protrude into the lumen of the nasal cavity and their axons project directly into the olfactory bulb. Hussar *et al*. have demonstrated structural differences in the blood-nerve barrier along these different portions of the ORN (36). In addition, the tight junction protein occludin was found to be absent from the nerve fiber bundles in the epithelium, which is a marked contrast to the blood-nerve barrier in typical peripheral nerves such as the sciatic nerve (36,37). We observed in our analysis that compounds with lower *ke0* (slower distribution to the site of action) in the olfaction assay tend to display an approximate 4 to 10-fold rightward shift in thermode assay potency relative to the potency in olfaction assay. It is reasonable to hypothesize that the blood-nerve barrier could be even more restrictive at the peripheral nerves in forearm than at the olfactory epithelium. Therefore, although the *ke0* parameter is associated with a hypothetical compartment in the PK-PD model, it may have physiological relevance with respect to the rate of compound penetration into the nerve tissue. The potential for different *ke0* parameter values should be considered when comparing PK-PD across different sites of action.

The established PK-PD model to quantitatively integrate *in vitro* potency, protein binding, and *in vivo* PK to predict *in vivo* target modulation and analgesic efficacy in the rhesus monkey provided a translational approach to inform potential efficacious human doses for candidate molecules. *In vitro* measurement of species-specific intrinsic potency against human NaV1.7 and human plasma protein binding can be used to predict target efficacious exposures at the site of action based on the IVIVC established in rhesus. However, translation of the *ke0* parameter from rhesus olfaction to human pain will need to carefully consider potential species and tissue differences in distribution to the site of action. A recent clinical study with a NaV1.7 inhibitor, GDC-0276, monitored reduced sense of smell (hyposmia) as a potential biomarker of on-target pharmacology (38). While a couple of incidents of hyposmia were reported, the authors conclude that due to a lack of an exposure-related pattern the findings do not support impaired sense of smell as a biomarker. However, the exposure required for antinociceptive efficacy has not yet been determined, and it is possible that the relatively low unbound plasma concentrations obtained in this study did not achieve sufficient target engagement at the site of action to inhibit olfaction or pain signal. The authors themselves acknowledge that further study will be needed to define the exposures required to achieve on-target pharmacodynamic effects.

## Conclusion

In conclusion, we have demonstrated the application of translational quantitative pharmacokinetic-pharmacodynamic modeling in the drug discovery stage to address key questions relevant to the ‘three pillars of survival’ and inform study design for preclinical efficacy studies. Parameterization of the model with appropriate species-specific input values, such as intrinsic potency, plasma protein binding, and PK along with consideration of key assumptions regarding drug distribution may enable prospective prediction of efficacious human dosing regimen.

## Electronic supplementary material

ESM 1 (PDF 162 kb)

ESM 2 (PDF 916 kb)

ESM 3 (PDF 512 kb)

## Abbreviations

fMRI: Functional magnetic resonance imaging
IM: Intramuscular
IV: Intravenous
IVIVC: *In vitro* to *in vivo* correlation
KO: Knock-out
PK-PD: Pharmacokinetics-pharmacodynamics
NaV1.7: Voltage-gated sodium ion channel isoform 1.7
NHP: Non-human primate
OB: Olfactory bulb
ORN: Olfactory receptor neuron
PD: Pharmacodynamic
PK: Pharmacokinetic
SC: Subcutaneous
UPLC: Ultra-high performance liquid chromatography
