# Supplementary material for: Application of Pharmacokinetic-Pharmacodynamic Modeling to Inform Translation of In Vitro NaV1.7 Inhibition to In Vivo Pharmacological Response in Non-human Primate
Source: Pharm Res. 2020 Sep 4;37(10):181. doi: 10.1007/s11095-020-02914-9 (PMC7473964; doi:10.1007/s11095-020-02914-9)
Supplement: Supplementary file 1 — (PDF 162 kb) [file 11095_2020_2914_MOESM1_ESM.pdf]

Online Resource 1

Journal: Pharmaceutical Research

Title: Application of pharmacokinetic-pharmacodynamic modeling to inform translation of *in vitro* NaV1.7 inhibition to *in vivo* pharmacological response in non-human primate

Authors: Jeanine E. Ballard[1], Parul Pall[2], Joshua Vardigan[2], Fuqiang Zhao[3], Marie A. Holahan[3], Richard Kraus[4], Yuxing Li[4], Darrell Henze[5], Andrea Houghton[5], Christopher S. Burgey[6], Christopher Gibson[1]

Affiliations: Merck & Co., Inc., Kenilworth, NJ USA

[1] Pharmacokinetics, Pharmacodynamics, and Drug Metabolism

[2] In Vivo Neuropharmacology

[3] MR-CT-US & Optical Imaging

[4] Neuronal Signaling

[5] Quantitative Biosciences

[6] Discovery Chemistry

Corresponding author: Jeanine E. Ballard

jeanine_ballard@merck.com

Supplementary Table S1. Study design details for fMRI olfaction assay

Test Compound	Bolus Dose (mg/kg)	Infusion Dose (mg/kg/hr)	Number of Animals
vehicle	0	0	6
1	1.5	7.5	6
2	4	5.5	5
3	0.32	2.8	6
	0.65	5.6	6
4	0.19	0.16	6
	0.75	0.88	6
5	0.23	2.1	6
	0.46	4.2	5
6	0.49	0.85	5
	0.98	1.7	4
	1.47	2.55	4
	1.67	3.33	1
	2.5	5	6
	4.41	7.65	5
	5	10	1
	8.82	15.3	5
7	0.32	4.21	4
	1.6	21	5
8	0.76	3.81	5
	2.3	11.5	5
9	0.31	1.66	4
10	0.6	9	2
	1.2	18	3
11	0.2	0.35	4

12	0.1	0.5	3
	0.5	2.5	3
13	0.14	0.35	2
	0.28	0.7	1
	0.42	1.05	5
	0.84	2.1	5
14	0.5	1	3
	1.5	3	3
	3	6	4
15	10	25	3
16	1	1.5	2
	3	5	2
17	0.6	0.6	3
18	0.6	1.4	2
	1.8	4.2	2
19	0.8	2.8	2
	2.5	8.5	1
20	5	15	5
21	0.2	0.2	3
	0.6	0.6	1
	1.8	1.8	1
22	0.3	0.3	3
	0.9	0.9	3
23	0.25	0.5	3
24	0.03	0.03	3
	0.07	0.07	6
	0.15	0.15	3

	0.5	0.5	3
25	0.4	0.4	1
	1.2	1.2	3
26	0.5	1	4
27	0.25	0.4	3
	0.8	1.2	3

Supplementary Table S2. Study design details for thermode assay

Test Compound	Subcutaneous Dose (mg/kg)	Number of Animals
6	0	7
	10	2
	20	7
12	0	8
	1	7
	2.5	7
	5	8
13	0	6
	0.5	6
	1	6
	2.5	6
	5	6
21	0	6
	1	6
	2	6
	5	6
22	0	7
	1	7
	3	7
	5	4
	10	3
24	0	7
	0.5	7
	1	7
	2	7

25	0	8
	2.5	8
	5	8
	10	8

Supplementary Table S3. Study design details for satellite PK studies

Test Compound	IV Bolus Dose (mg/kg)	IV Infusion Dose (mg/kg/hr)	Number of Animals
3	2		2
6		5	3
		15	3
12	0.5		3
13		2	3
		8	3
14	0.05		2
16	1		3
18	0.05		2
19	0.05		2
21	0.5		3
22	0.05		2
24	0.05		2
25	0.05		2
27	0.2		2
