# Supplementary material for: Application of Pharmacokinetic-Pharmacodynamic Modeling to Inform Translation of In Vitro NaV1.7 Inhibition to In Vivo Pharmacological Response in Non-human Primate
Source: Pharm Res. 2020 Sep 4;37(10):181. doi: 10.1007/s11095-020-02914-9 (PMC7473964; doi:10.1007/s11095-020-02914-9)

Online Resource 2

Journal: Pharmaceutical Research

Title: Application of pharmacokinetic-pharmacodynamic modeling to inform translation of *in vitro* NaV1.7 inhibition to *in vivo* pharmacological response in non-human primate

Authors: Jeanine E. Ballard[1], Parul Pall[2], Joshua Vardigan[2], Fuqiang Zhao[3], Marie A. Holahan[3], Richard Kraus[4], Yuxing Li[4], Darrell Henze[5], Andrea Houghton[5], Christopher S. Burgey[6], Christopher Gibson[1]

Affiliations: Merck & Co., Inc., Kenilworth, NJ USA

[1] Pharmacokinetics, Pharmacodynamics, and Drug Metabolism

[2] In Vivo Neuropharmacology

[3] MR-CT-US & Optical Imaging

[4] Neuronal Signaling

[5] Quantitative Biosciences

[6] Discovery Chemistry

Corresponding author: Jeanine E. Ballard

jeanine_ballard@merck.com

Supplementary Figure S4. Visual predictive check of model predicted versus observed plasma concentration over time for each compound in satellite PK studies

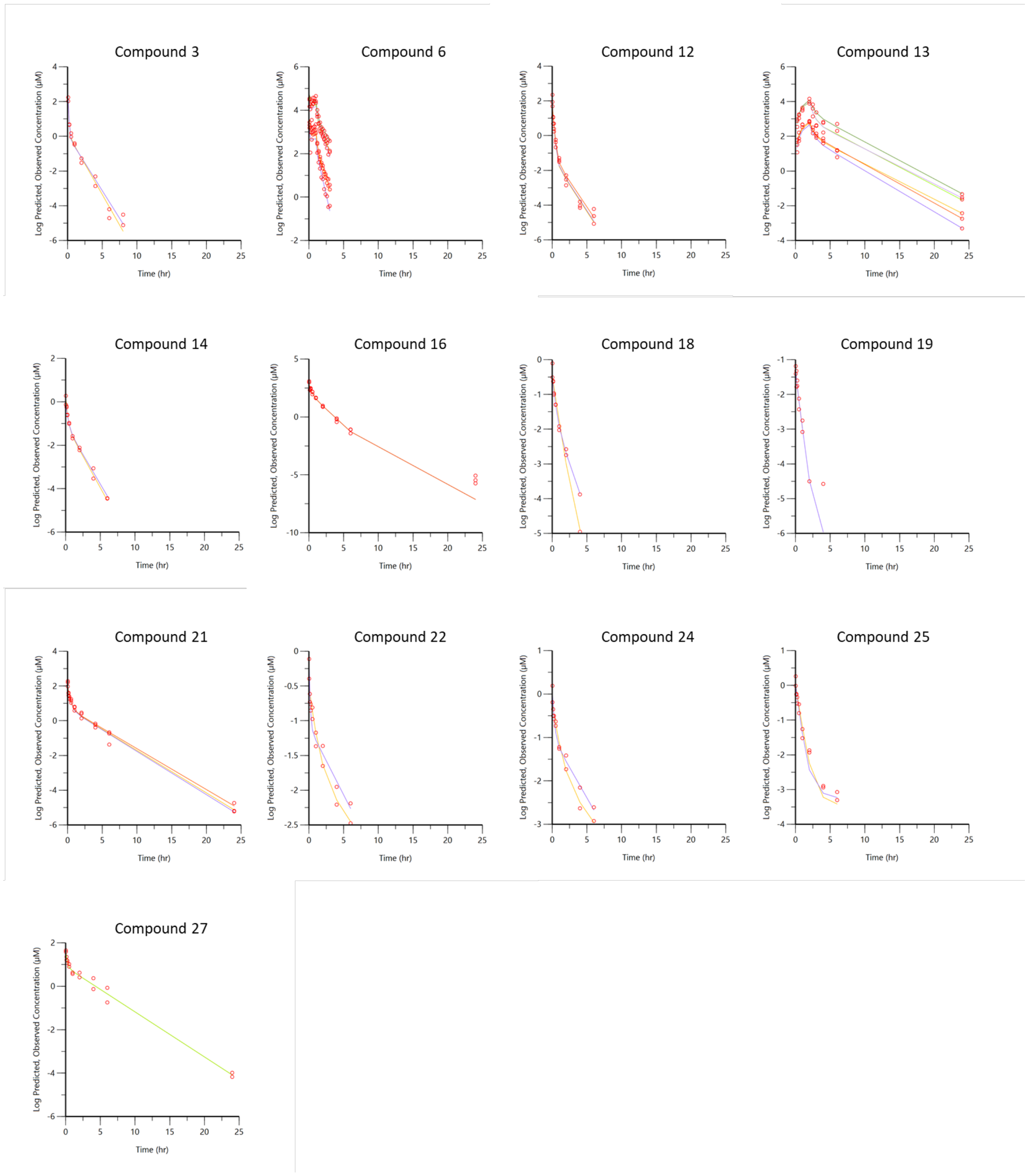

Supplementary Figure S5. Visual predictive check of simulated versus observed plasma concentration over time for each compound in fMRI PK studies

(a) Compounds with acceptable simulation of IV infusion PK profile

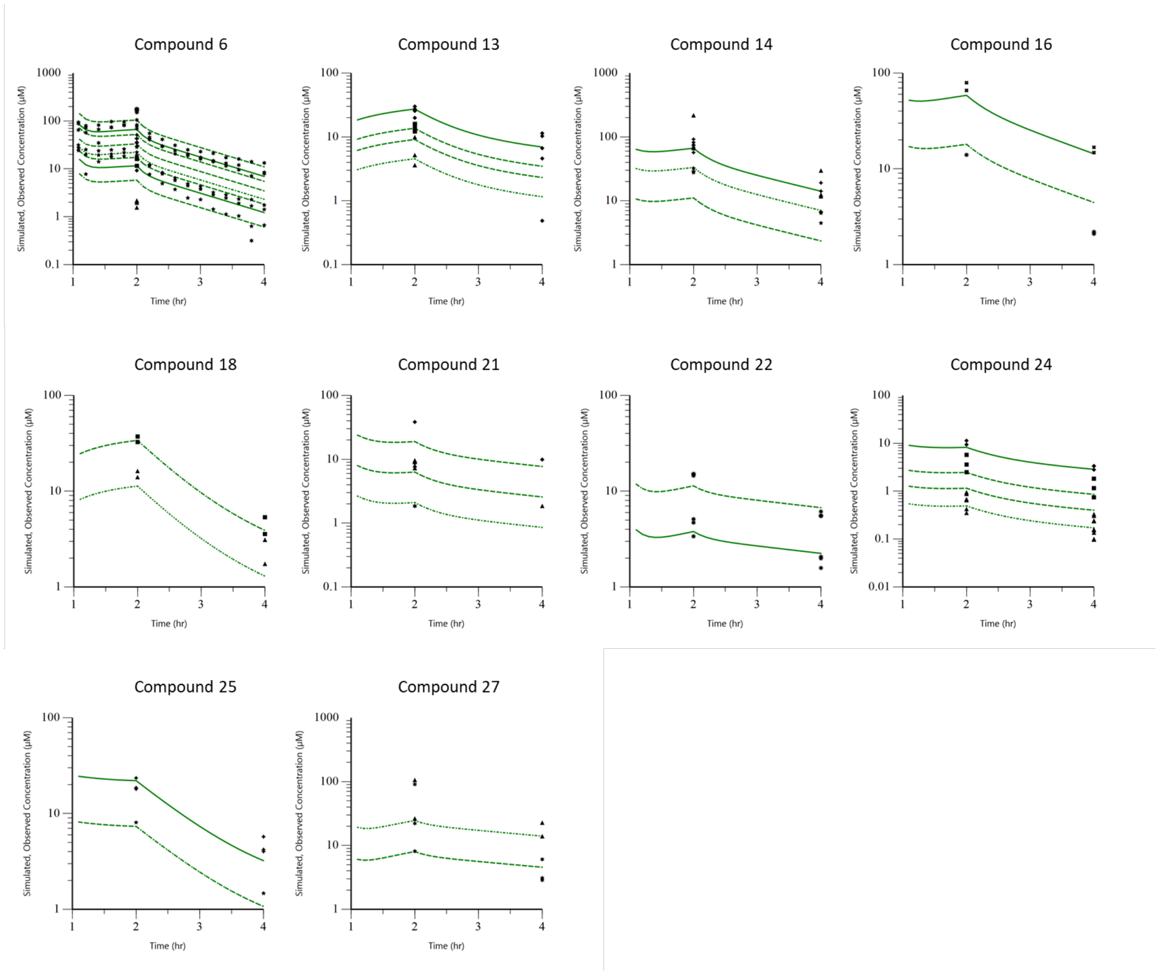

(b) Compounds with unacceptable simulation of IV infusion PK profile

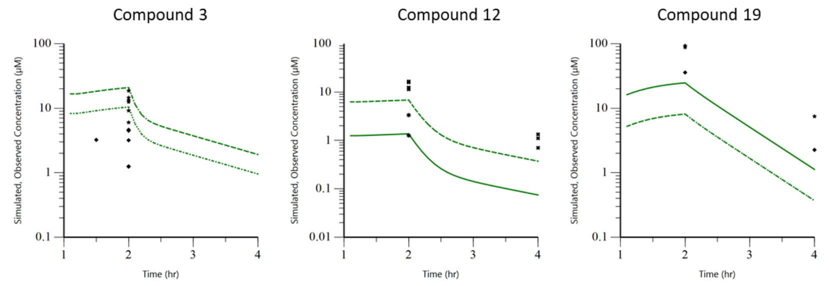

Supplementary Figure S6. Visual predictive check of effect compartment PK-PD model predicted versus observed percent inhibition of fMRI signal over time for each compound with washout data in the fMRI assay

Compound 6

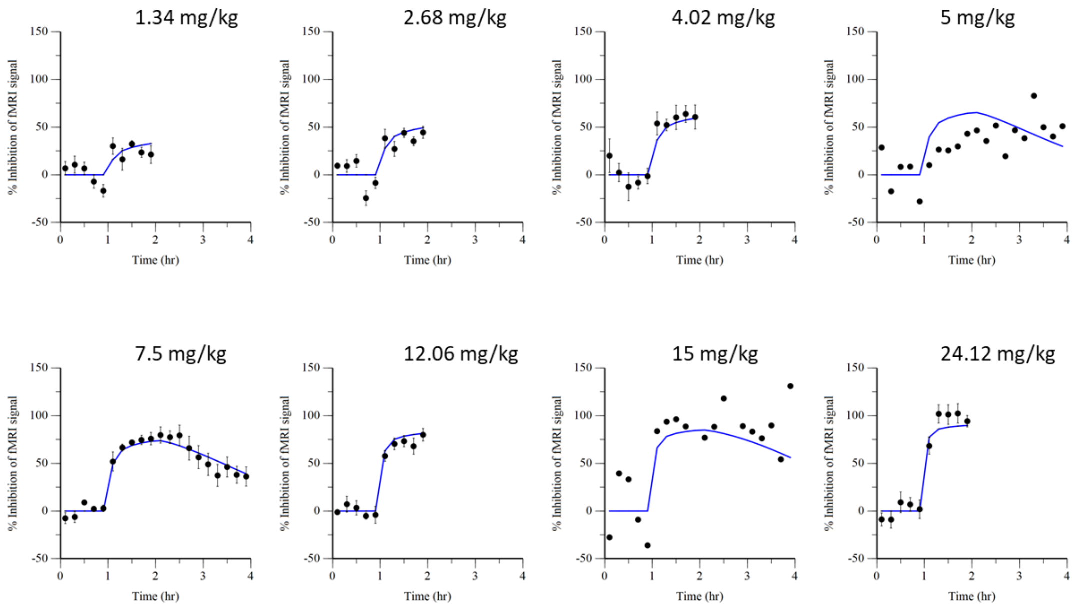

Compound 13

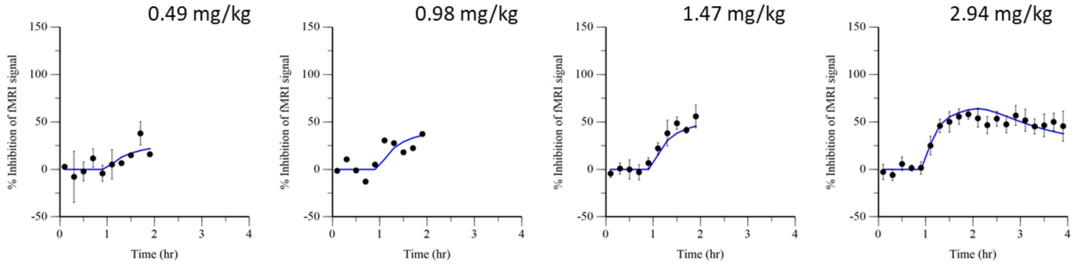

Compound 14

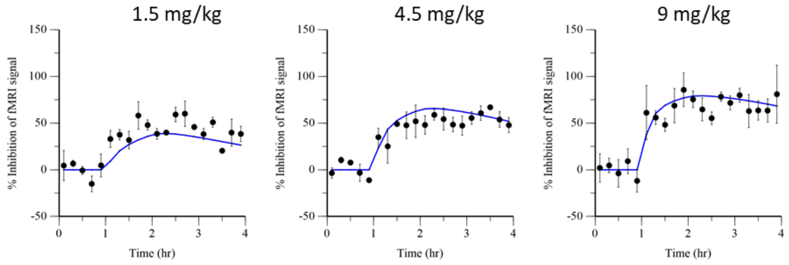

Compound 16

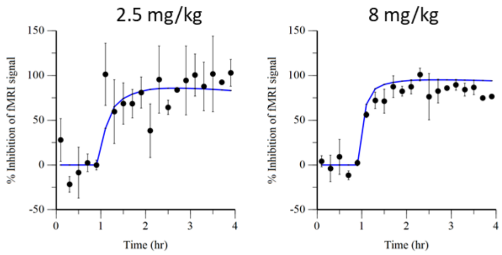

Compound 18

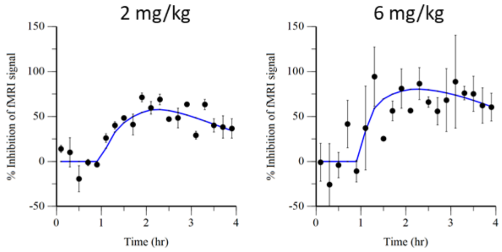

Compound 21

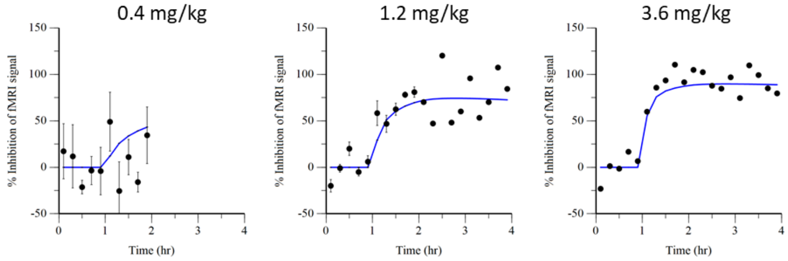

Compound 22

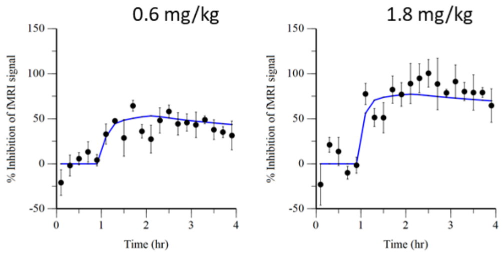

Compound 24

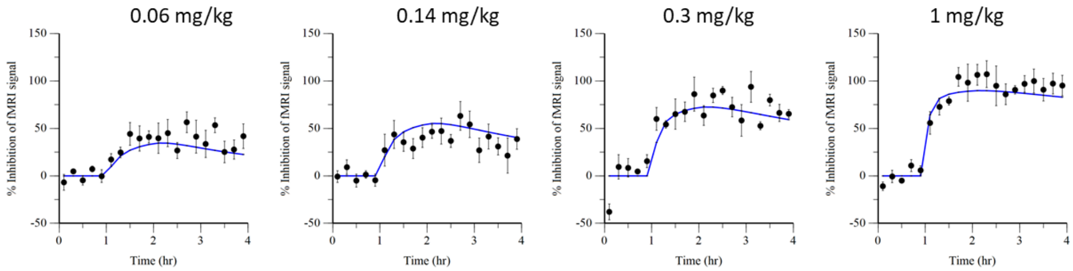

Compound 25

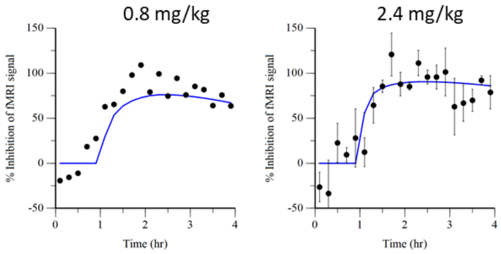

Compound 27

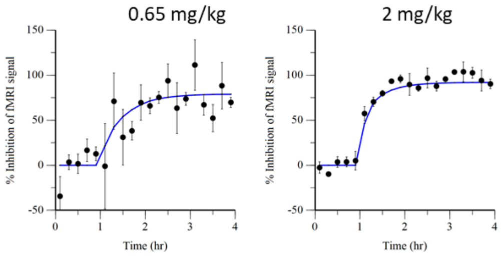

Supplement: Supplementary file 2 — (PDF 916 kb) [file 11095_2020_2914_MOESM2_ESM.pdf]
