# Supplementary material for: Application of Pharmacokinetic-Pharmacodynamic Modeling to Inform Translation of In Vitro NaV1.7 Inhibition to In Vivo Pharmacological Response in Non-human Primate
Source: Pharm Res. 2020 Sep 4;37(10):181. doi: 10.1007/s11095-020-02914-9 (PMC7473964; doi:10.1007/s11095-020-02914-9)

Online Resource 3

Journal: Pharmaceutical Research

Title: Application of pharmacokinetic-pharmacodynamic modeling to inform translation of *in vitro* NaV1.7 inhibition to *in vivo* pharmacological response in non-human primate

Authors: Jeanine E. Ballard[1], Parul Pall[2], Joshua Vardigan[2], Fuqiang Zhao[3], Marie A. Holahan[3], Richard Kraus[4], Yuxing Li[4], Darrell Henze[5], Andrea Houghton[5], Christopher S. Burgey[6], Christopher Gibson[1]

Affiliations: Merck & Co., Inc., Kenilworth, NJ USA

[1] Pharmacokinetics, Pharmacodynamics, and Drug Metabolism

[2] In Vivo Neuropharmacology

[3] MR-CT-US & Optical Imaging

[4] Neuronal Signaling

[5] Quantitative Biosciences

[6] Discovery Chemistry

Corresponding author: Jeanine E. Ballard

jeanine_ballard@merck.com

Supplementary Figure S7. Simulated versus observed plasma concentration and nociceptive response over time in the NHP Thermode assay. Cp (black lines) are simulated concentrations in the plasma, Ce (blue lines) are simulated concentrations in the effect compartment.

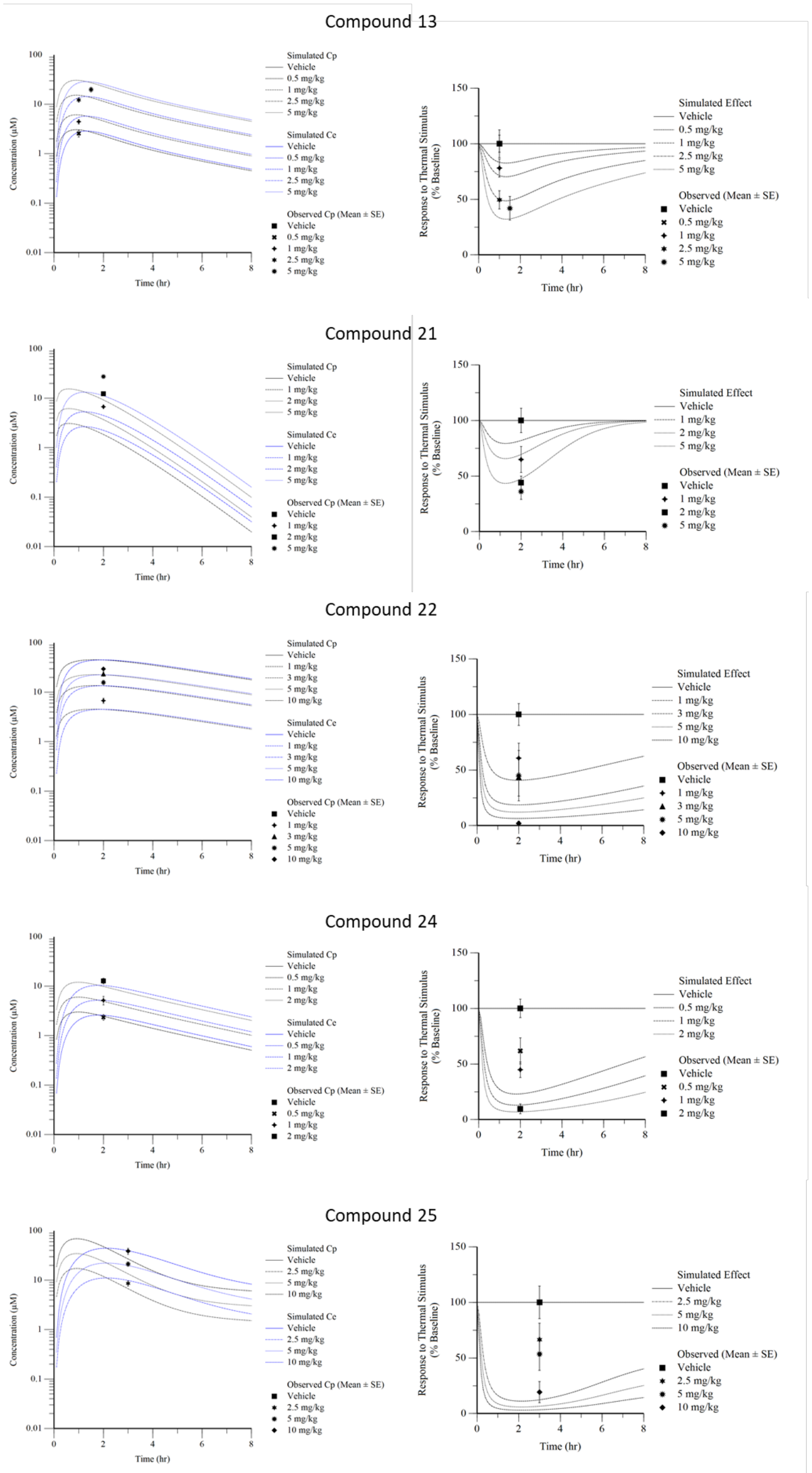

Supplement: Supplementary file 3 — (PDF 512 kb) [file 11095_2020_2914_MOESM3_ESM.pdf]
